# Construction of a circRNA-miRNA-mRNA network based on competitive endogenous RNA reveals the function of circRNAs in osteosarcoma

**DOI:** 10.1186/s12935-020-1134-1

**Published:** 2020-02-10

**Authors:** Yu Qiu, Chao Pu, Yanchao Li, Baochuang Qi

**Affiliations:** 1 Department of Orthopaedics, First People’s Hospital of Yibin, Yibin, 644000 Sichuan China; 2 Second Clinical Medical College of North Sichuan Medical College, Nanchong Central Hospital, Nanchong, 637000 Sichuan China

**Keywords:** Osteosarcoma, CircRNA, GEO, TARGET, Network

## Abstract

**Background:**

Osteosarcoma (OS) is a common primary malignant bone tumour. Growing evidence suggests that circular RNAs (circRNAs) are closely related to the development of tumours. However, the function of circRNAs in OS remains unknown. Here, we aimed to determine the regulatory mechanisms of circRNAs in OS.

**Methods:**

The expression profiles of OS circRNA (GSE96964), microRNA (GSE65071) and mRNA (GSE33382) were downloaded from the Gene Expression Omnibus (GEO) database to identify differentially expressed circRNAs, miRNAs and mRNAs in OS. A ceRNA network was constructed based on circRNA-miRNA pairs and miRNA-mRNA pairs. MRNAs with significant prognostic differences were identified by the TARGET database in the network. Functional and pathway enrichment analyses were performed, and interactions between proteins were predicted using Cytoscape. Gene Ontology (GO) and Kyoto Encyclopedia of Genes and Genomes (KEGG) analyses were performed to elucidate the possible functions of these differentially expressed circRNAs.

**Results:**

A total of 15 downregulated circRNAs, 136 upregulated miRNAs and 52 downregulated mRNAs were identified in OS. Finally, a circRNA-miRNA-mRNA network was constructed in OS based on 14 circRNAs, 24 miRNAs, and 52 mRNAs. GO and KEGG pathway analyses suggested that the mRNAs in the network may be involved in the pathogenesis and progression of OS. Four mRNAs identified by the TARGET database were significantly associated with OS survival prognosis. A circRNA-miRNA-mRNA subnetwork was constructed based on these four mRNAs.

**Conclusion:**

Our results provide a deeper understanding of the regulatory mechanisms by which circRNAs compete for endogenous RNAs in OS.

## Background

Osteosarcoma (OS) is one of the most common bone malignancies [1]. It mainly occurs during adolescence, and its incidence decreases with age [2, 3]. At present, the treatment of OS mainly involves surgery and adjuvant chemotherapy, but the prognosis of patients is still not optimistic [4, 5]. Studies have found that the occurrence and development of OS is closely related to gene mutations and gene expression disorders [6–8]. Currently, gene-targeted therapy has been widely studied in various cancers and may be a great potential treatment strategy [9–11]. Therefore, studying the molecular mechanism of OS regarding its occurrence and development processes and finding effective molecular targets are crucial for the diagnosis and treatment of OS.

Circular RNAs (circRNAs) are a new class of non-coding RNAs with covalently closed loop structures [12, 13]. Due to the lack of a 5′ cap structure and a 3′ poly (A) tail, circRNAs are resistant to exonuclease and are more stable than linear RNAs [14]. A growing body of research indicates that circRNAs play an important regulatory role in the development of cancer in vivo. Zhou et al. found that by sponging miR-203, circRNA-0008717 inhibits the proliferation and invasion of OS cells [15]. Song et al. found that hsa_circ_0001564 acts as a miR-29c-3p sponge to cause tumourigenesis and may serve as a potential biomarker for OS [16]. In addition, a related study found that circRNA CDR1as/miR-7 signalling promotes tumour growth in OS, with potential therapeutic and diagnostic value [17]. These studies indicate that circRNAs play an important role in the development and progression of OS.

**Fig. 1:**
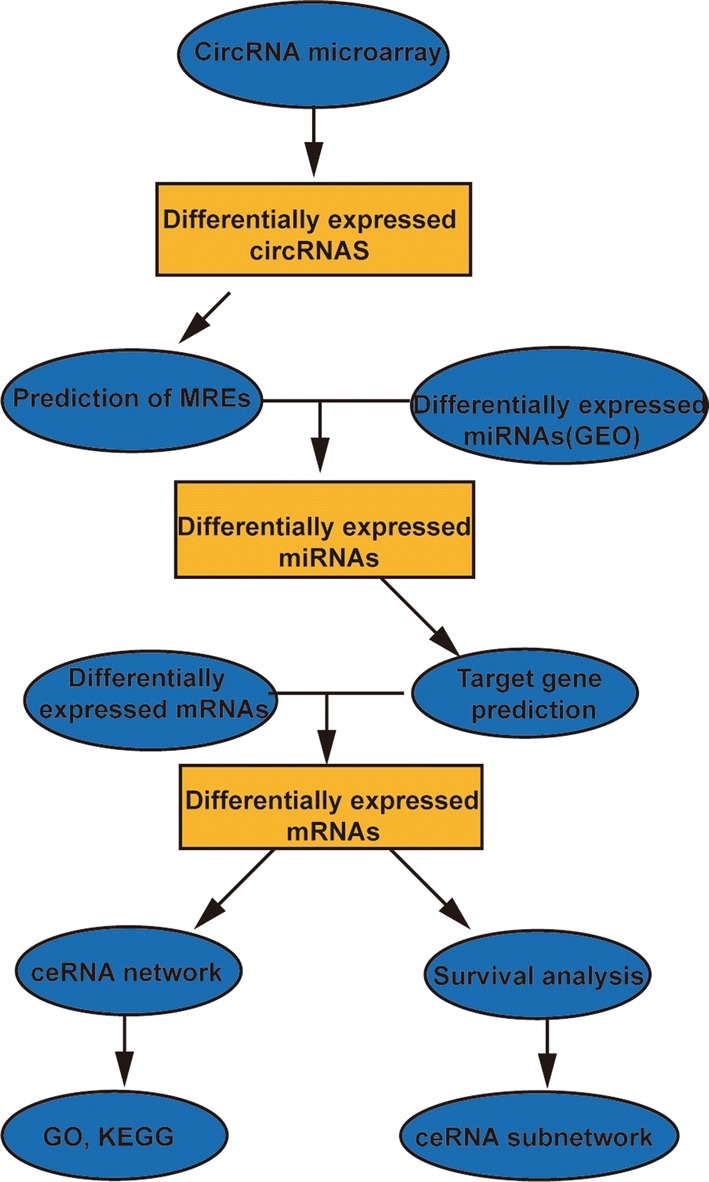
Flow chart of the approach utilized in the present study

In this study, we collected the expression profiles of circRNAs, miRNAs and mRNAs in OS from the Gene Expression Omnibus (GEO) database and the TARGET database. Differentially expressed circRNAs, miRNAs and mRNAs were identified. CircRNA-miRNA pairs and miRNA-mRNA pairs were screened, and a circRNA-miRNA-mRNA network was constructed. The functional pathways of the mRNAs in this network were analysed by Gene Ontology (GO) and Kyoto Encyclopedia of Genes and Genomes (KEGG) functional enrichment. To better understand the pathogenesis of OS, TARGET data were used to screen mRNAs related to OS prognosis, and a circRNA-miRNA-mRNA subnetwork related to OS prognosis was constructed. Our research provides new insights into the mechanisms of the occurrence and development of OS. The flowchart for this procedure is shown in Fig. 1.

## Methods

### Data acquisition

GEO (http://www.ncbi.nlm.nih.gov/geo) is a public functional genomic database. Three datasets (GSE96964, GSE65071, and GSE33382; analysed on Agilent-069978 Arraystar Human CircRNA microarray V1, Exiqon human V3 microRNA PCR panel I + II, and Illumina human-6 v2.0 expression beadchip, respectively) were downloaded from GEO. The probes were converted to the corresponding gene symbols based on the annotation information on the platform. The GSE96964 dataset contained a human osteoblast cell line (hFOB1.19) and 7 human osteosarcoma cell lines (U2OS, MTX300, HOS, MG63,143B, ZOS, and ZOSM). The GSE65071 dataset contained 15 healthy people and 20 patients with osteosarcoma. The GSE33382 dataset contained 3 human osteoblasts and 83 osteosarcoma samples. The TARGET database (https://ocg.cancer.gov/programs/target) is a database of childhood tumours that seeks to determine molecular changes in the development and progression of refractory childhood cancer using a comprehensive genomic approach. This database contains gene expression profiles and clinical information for patients with osteosarcomas. We obtained 101 osteosarcoma samples with complete clinical information from the TARGET database. Approval by the Ethics Committee was not necessary because all data were collected from publicly available databases (GEO and TARGET).

### Identification of differential expression of circRNAs, miRNAs and mRNAs

The raw data were processed by background correction and normalization by using the affy package of R/Bioconductor. The Limma and edgeR packages were used to identify differentially expressed circRNAs, miRNAs and mRNAs between normal samples and tumour samples. Log [fold change (FC)] > 2 and adj. *P* value < 0.05 were considered to indicate a statistically significant difference.

### Construction of the ceRNA network

Information about circRNAs is available on the circBase website (http://www.circbase.org/). The cancer-specific circRNA database (CSCD) (https://gb.whu.edu.cn/CSCD/) was used to predict target miRNAs. These target miRNAs were further screened by miRNAs differentially expressed in GSE65071. Then, the miRDB, TargetScan and miRTarBase databases were employed to predict specific miRNAs. Only mRNAs recognized by the 3 databases were considered to be candidate targets and were intersected with the identified differentially expressed mRNAs to identify the differentially expressed mRNAs targeted by the differentially expressed miRNAs. Finally, the circRNA-miRNA-mRNA regulatory network was constructed by screening the circRNA-miRNA and miRNA-mRNA groups. The data were visualized using Cytoscape 3.7.1.

### GO and KEGG functional enrichment analysis

Database for Annotation, Visualization and Integrated Discovery (DAVID; version 6.7; http://david.ncifcrf.gov) is an online bioinformatics database that integrates biological data and analysis tools and provides gene annotation information and protein data. KEGG is a database resource for understanding advanced functions and biological systems from large-scale molecular data generated by high-throughput experimental techniques. GO is a major bioinformatics tool for annotating genes and analysing their biological processes (BPs), molecular functions (MFs) and cellular components (CCs). To determine the function of differentially expressed mRNAs in the network, analysis was performed using DAVID. An adj. P-value < 0.05 was considered to indicate a statistically significant difference.

### Survival analysis

To analyse the mRNAs selected from the ceRNA network, the OS mRNA expression profile data and clinical sample data in the TARGET database were downloaded. The overall survival analysis was performed using Kaplan–Meier curves. The log-rank test was used for statistical analysis. The threshold for survival prognosis significance was a P-value < 0.05. A subnetwork was constructed based on these mRNAs.

## Results

### Identification of differentially expressed circRNAs, miRNAs and mRNAs

**Fig. 2:**
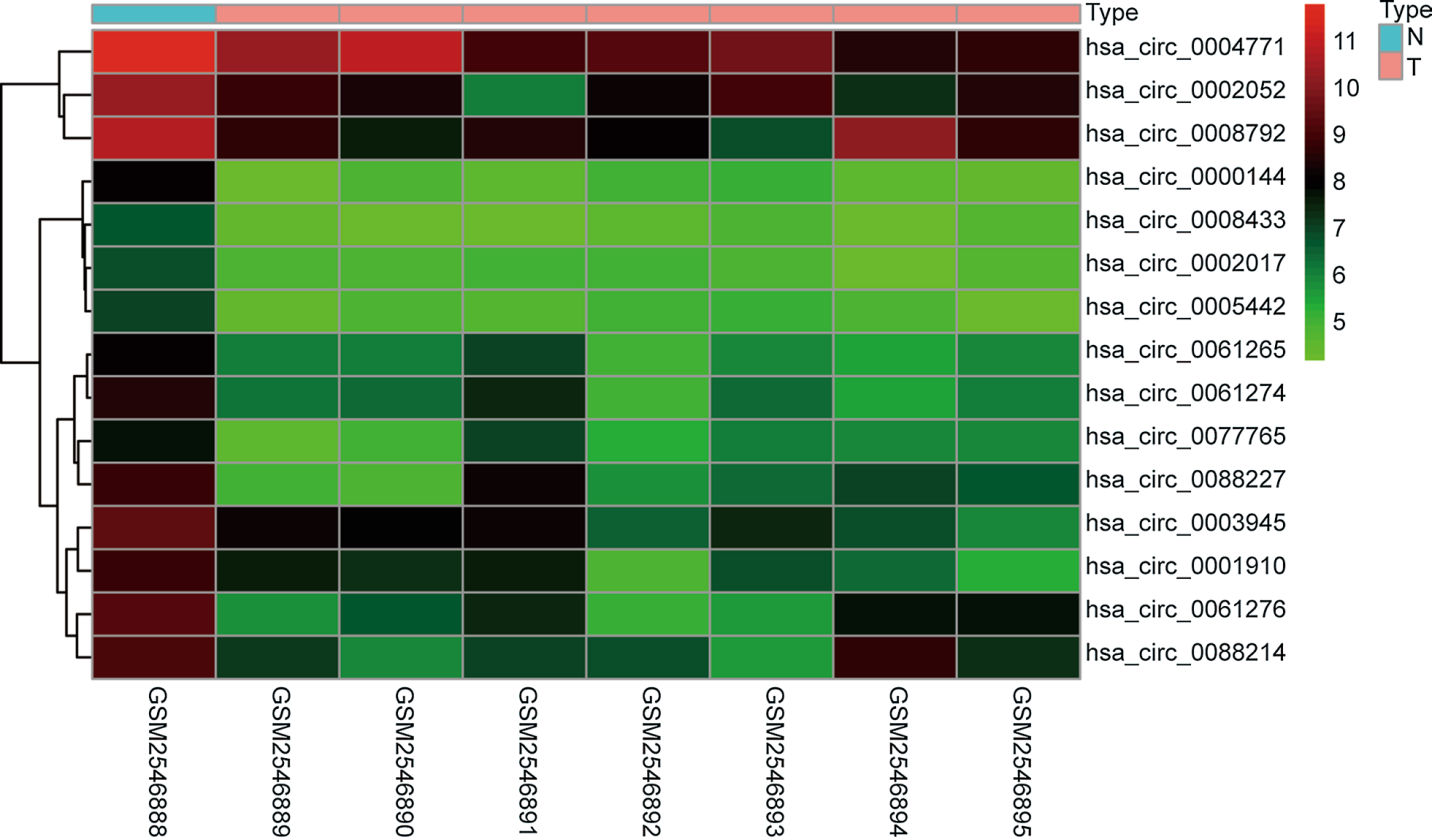
Heatmap of the fifteen differentially expressed circRNAs in the eight microarray datasets. By analysing the circRNA dataset, fifteen differentially expressed circRNAs were identified (|log 2 (fold change (FC))| > 2 and an adjusted P value < 0.05 were considered)

**Fig. 3:**
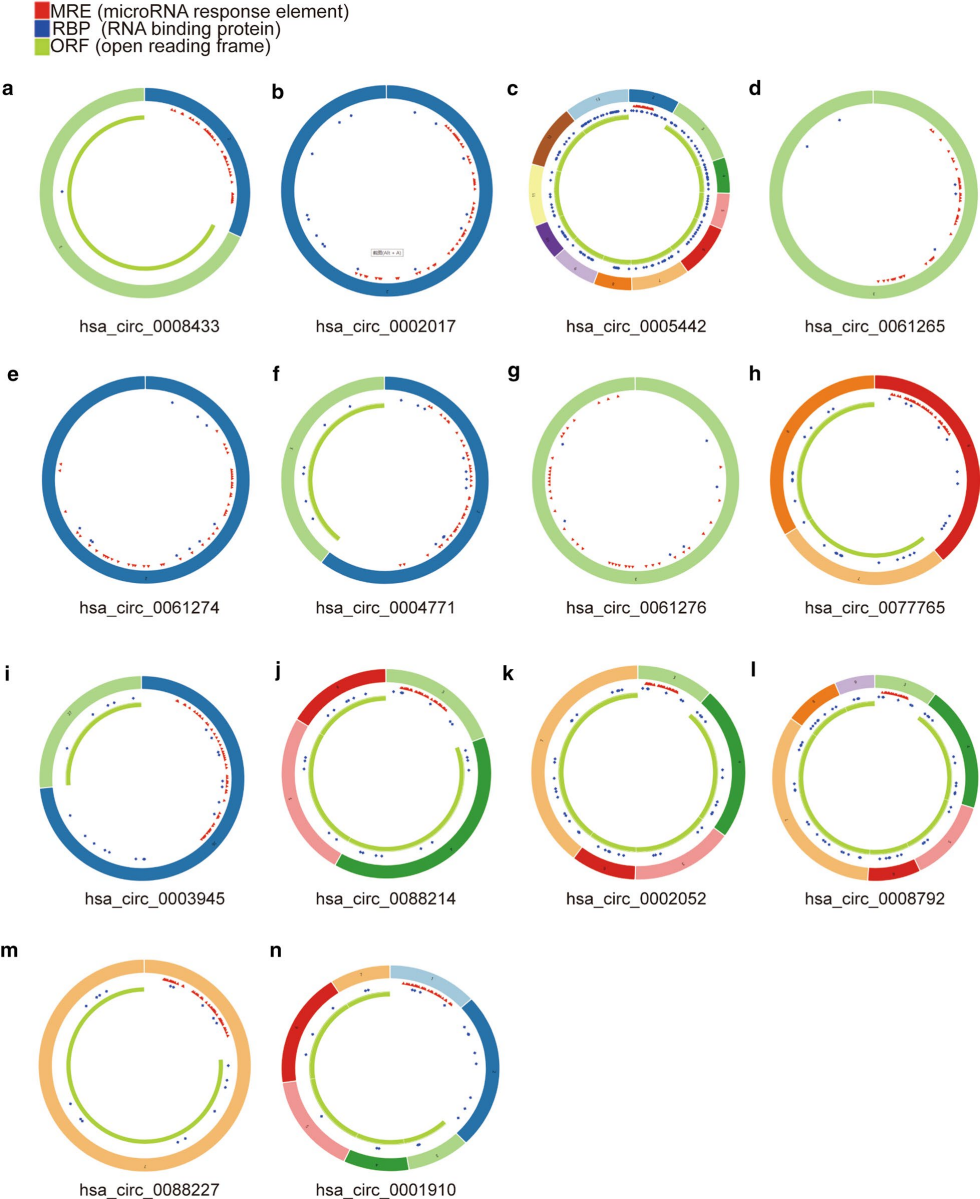
Structural patterns of the fourteen circRNAs. **a**–**n** A total of 14 circRNA structures were obtained from the circRNA website. Red represents the position where the miRNA may bind, blue represents the position where the protein may bind, and yellow represents the open reading frame

**Fig. 4:**
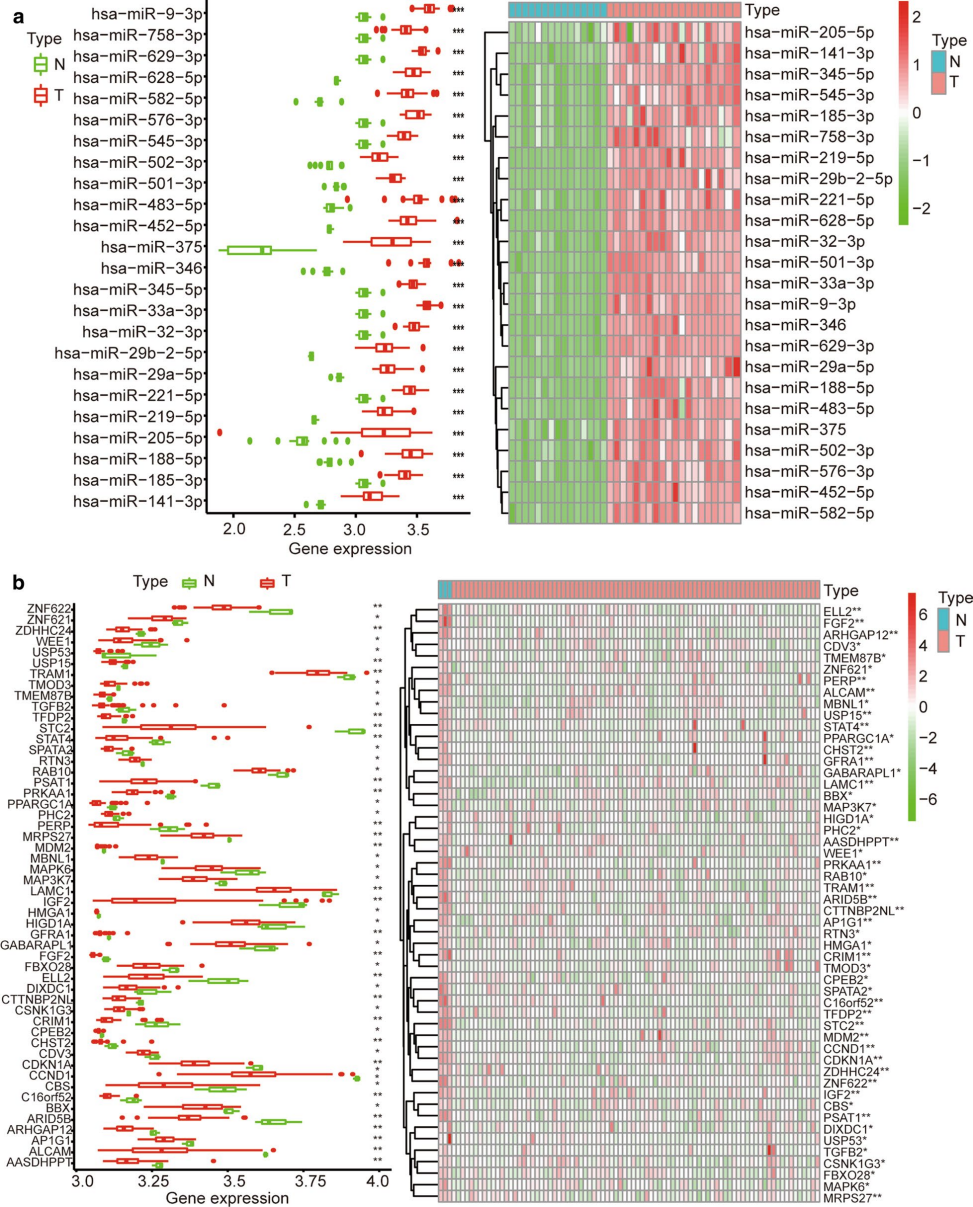
The differentially expressed miRNAs and mRNAs in the microarray datasets. **a** Twenty-four miRNAs were obtained from the intersection of miRNAs predicted from the website and the differentially expressed miRNAs identified in the miRNA dataset (GEO dataset). The expression levels of 24 miRNAs in the miRNA dataset are shown in **a**. **b** A total of 52 differentially expressed mRNAs were obtained by analysing the mRNA dataset and predicting the targeted binding of genes by the 24 miRNAs. The expression levels of these 52 mRNAs in the mRNA dataset are shown in **b**. (|log 2 (fold change (FC))| > 2, and an adjusted P value < 0.05 were the cut-off criteria). *P < 0.05, **P < 0.01, ***P < 0.001

The basic information regarding the expression of circRNAs, miRNAs and mRNAs in OS and control tissues in the three microarray datasets (GSE96964, GSE65071 and GSE33382) is listed in Table 1. In the circRNA expression profile data, a total of 15 circRNAs were downregulated in OS (Fig. 2, Additional file 1: Figure S1). The basic information for the 15 circRNAs is listed in Table 2. One of the circRNAs (hsa_circ_0000144) was not found in the CSCD database. From the CSCD database, we predicted that 503 miRNAs might target these 14 circRNAs (Additional file 2: Table S1). The basic structural pattern of the 14 circRNAs is shown in Fig. 3. In the miRNA expression profile data, a total of 136 miRNAs were upregulated in OS (Additional file 3: Table S2). Twenty-four intersecting miRNAs were obtained and are shown in Additional file 4: Table S3 (Fig. 4a). A total of 324 potential target genes (Additional file 5: Table S4) were predicted by the TargetScan, miRTarBase and miRDB databases. A total of 52 mRNAs were downregulated in OS in the mRNA expression profile data (Additional file 6: Table S5). There were 52 intersecting mRNAs (Fig. 4b), as shown in Additional file 7: Table S6.

**Table 1:** Basic information of the 4 microarray datasets from GEO and TARGET

Data source	Platform	Series	Sample size (T/N)
CircRNA	GPL19978	GSE96964	7/1
miRNA	GPL19631	GSE65071	20/15
mRNA	GPL10295	GSE33382	84/3
mRNA	TARGET	None	101/0

**Table 2:** Basic characteristics of the fifteen differentially expressed circRNAs

CircRNA ID	Position	Genomic length	Strand	Best transcript	Gene symbol
hsa_circ_0000144	chr1:160472466–160472794	328	+	NM_052931	SLAMF6
hsa_circ_0008433	chr1:66378927–66384518	5591	+	NM_001037341	PDE4B
hsa_circ_0002017	chr2:36623756–36623930	174	+	NM_016441	CRIM1
hsa_circ_0005442	chr2:36623756–36749456	125,700	+	NM_016441	CRIM1
hsa_circ_0061265	chr21:15456270–15456465	195	+	None	None
hsa_circ_0061274	chr21:16386664–16386787	123	−	NM_003489	NRIP1
hsa_circ_0004771	chr21:16386664–16415895	29,231	−	NM_003489	NRIP1
hsa_circ_0061276	chr21:16415815–16415895	80	−	NM_003489	NRIP1
hsa_circ_0077765	chr6:125366356–125398004	31,648	+	NM_152553	RNF217
hsa_circ_0003945	chr9:33953282–33956144	2862	−	NM_018449	UBAP2
hsa_circ_0088214	chr9:118969734–118989831	20,097	+	NM_002581	PAPPA
hsa_circ_0002052	chr9:118969734–118997916	28,182	+	NM_002581	PAPPA
hsa_circ_0008792	chr9:118969734–119033695	63,961	+	NM_002581	PAPPA
hsa_circ_0088227	chr9:118997417–118997916	499	+	NM_002581	PAPPA
hsa_circ_0001910	chrX:10031484–10066619	35,135	+	NM_015691	WWC3

### Construction of the ceRNA network

**Fig. 5:**
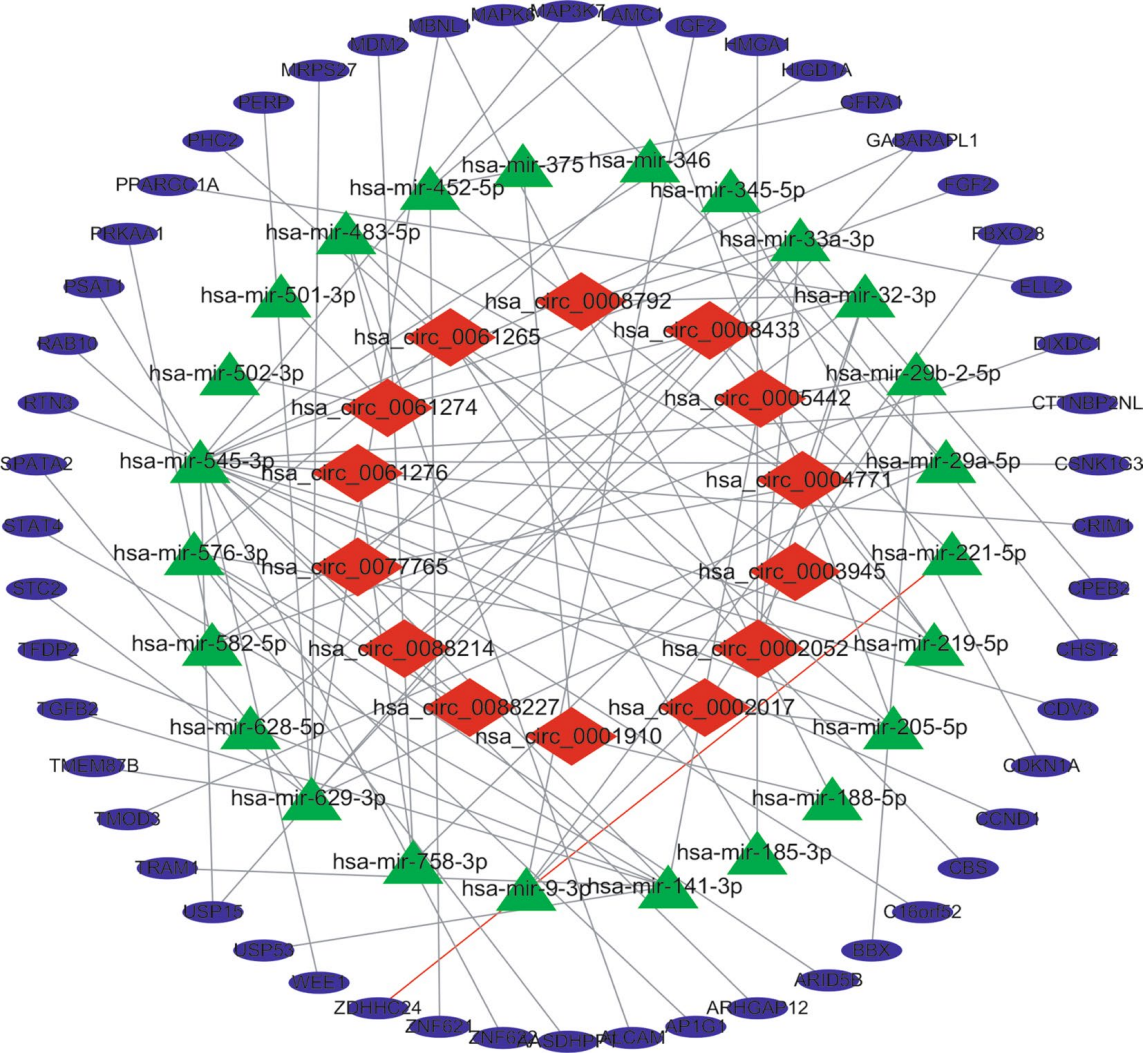
CeRNA network of circRNA-miRNA-mRNA interactions in OS. Red indicates circRNAs, green indicates miRNAs, and blue indicates mRNAs

We used 14 circRNAs, 24 miRNAs and 52 mRNAs in Cytoscape 3.7.1 to construct a circRNA-miRNA-mRNA visualization network (Fig. 5).

### Functional and pathway enrichment analysis

**Fig. 6:**
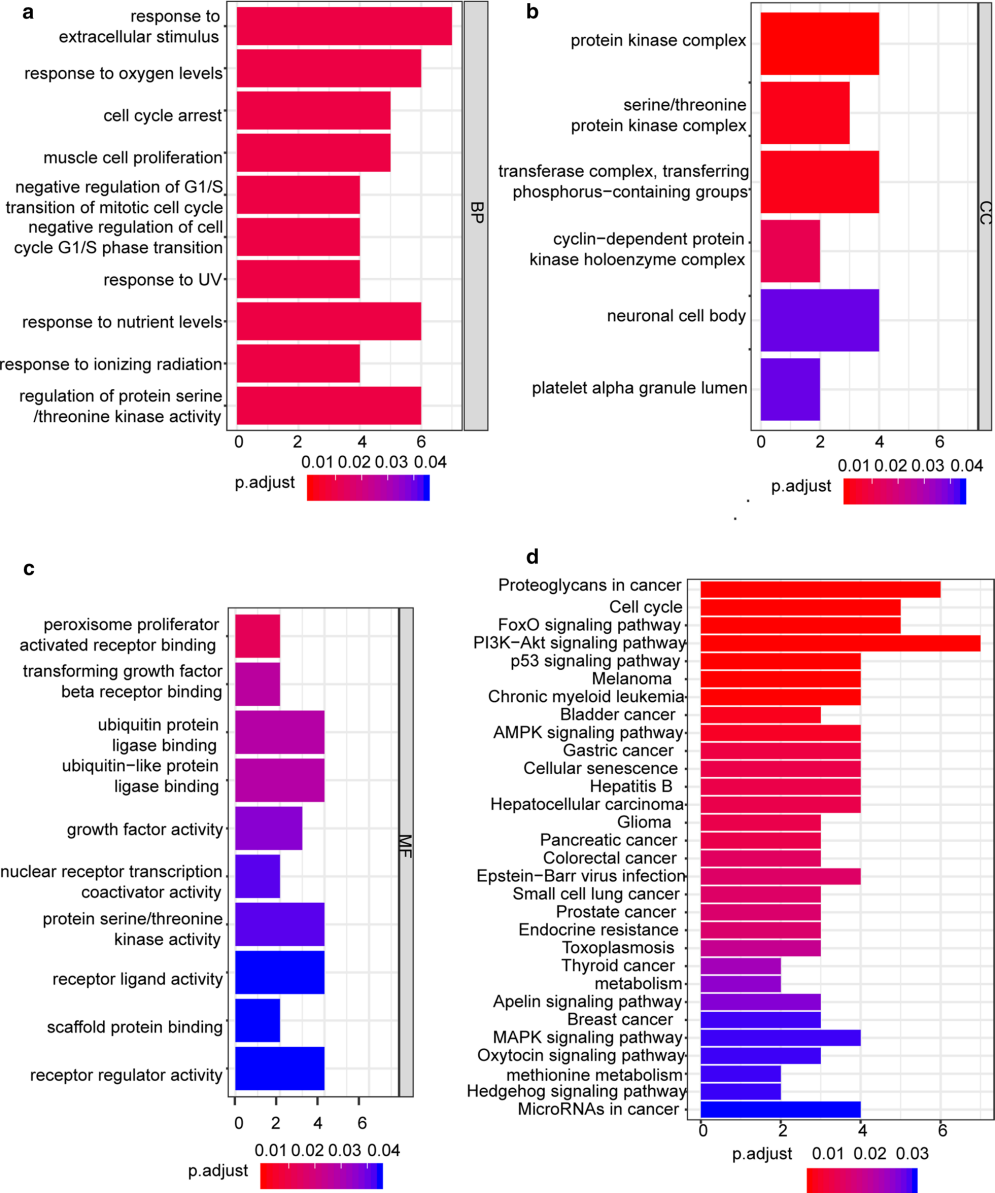
GO and KEGG functional enrichment analyses of the differentially expressed mRNAs in the ceRNA network. **a** The enrichment of biological processes. **b** The enrichment of cellular components. **c** The enrichment of molecular functions. **d** The enrichment analysis of the KEGG pathways. A P < 0.05 was considered to indicate a statistically significant difference

The results indicated that the identified mRNAs were mainly enriched in ‘response to extracellular stimulus’, ‘response to oxygen levels’, ‘response to nutrient levels’ and ‘regulation of protein serine/threonine kinase activity’ (BPs) (Fig. 6a); ‘protein kinase complex’, ‘transferase complex, transferring phosphorus-containing groups’ and ‘neuronal cell body’ (CCs) (Fig. 6b); and ‘ubiquitin protein ligase binding’ and ‘ubiquitin-like protein ligase binding’ (MFs) (Fig. 6c). KEGG pathway analysis revealed strong enrichment in the ‘PI3K-Akt signaling pathway’, ‘Proteoglycans in cancer’, ‘Cell cycle’ and ‘FoxO signaling pathway’ (Fig. 6d).

### Survival analysis and subnetwork construction of related mRNAs

**Fig. 7:**
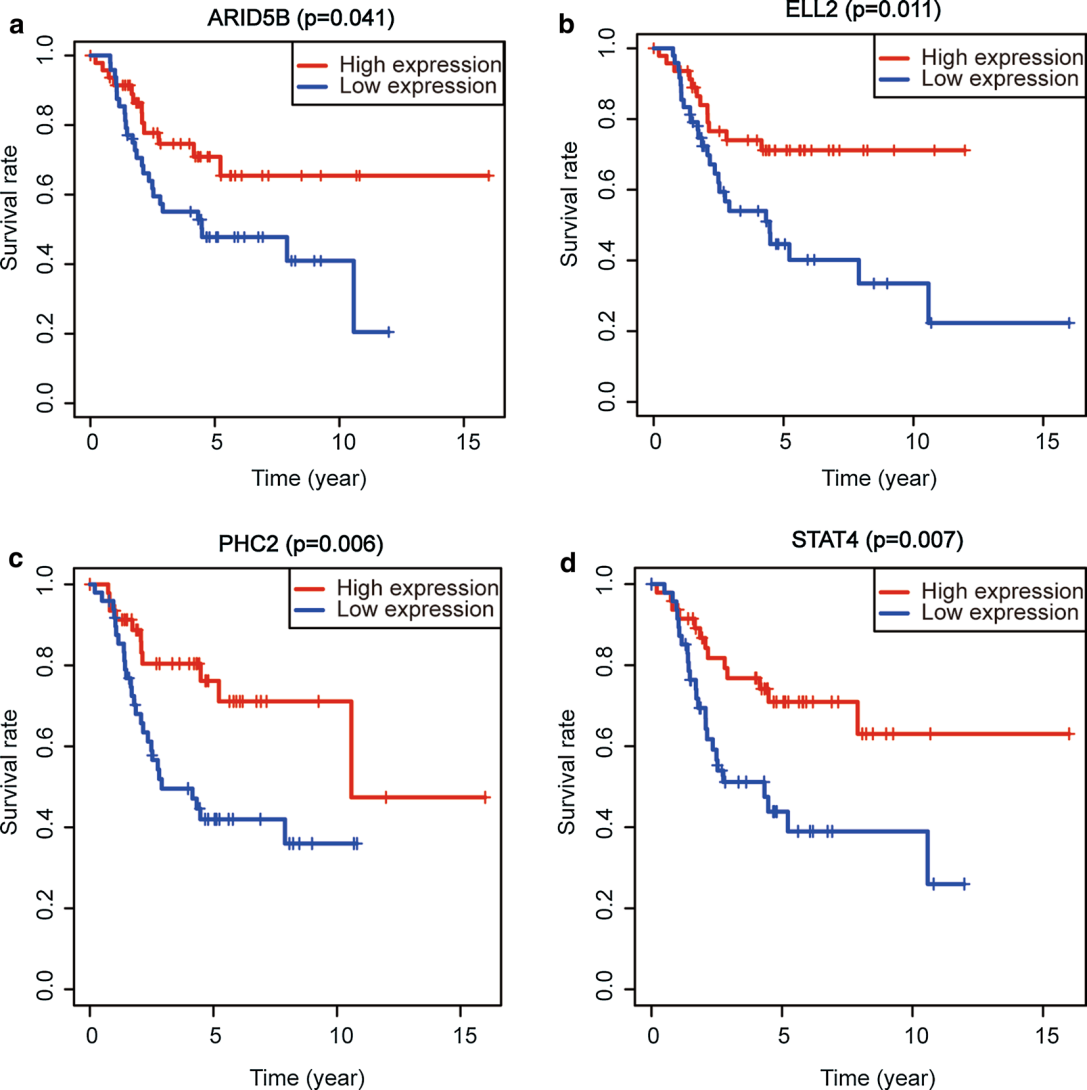
Survival analysis of the differentially expressed mRNAs in the ceRNA network. The TARGET database was used to analyse the survival prognosis of 52 mRNAs in osteosarcoma patients. **a** The ARID5B gene high expression group in osteosarcoma samples had a good prognosis compared with the low expression group (P = 0.041). **b** The ELL2 gene high expression group had a good prognosis compared with the low expression group (P = 0.011). **c** The PHC2 gene high expression group had a good prognosis compared with the low expression group (P = 0.006). **d** The STAT4 gene high expression group had a good prognosis compared with the low expression group (P = 0.007). (P value < 0.05 was the cut-off criterion)

**Fig. 8:**
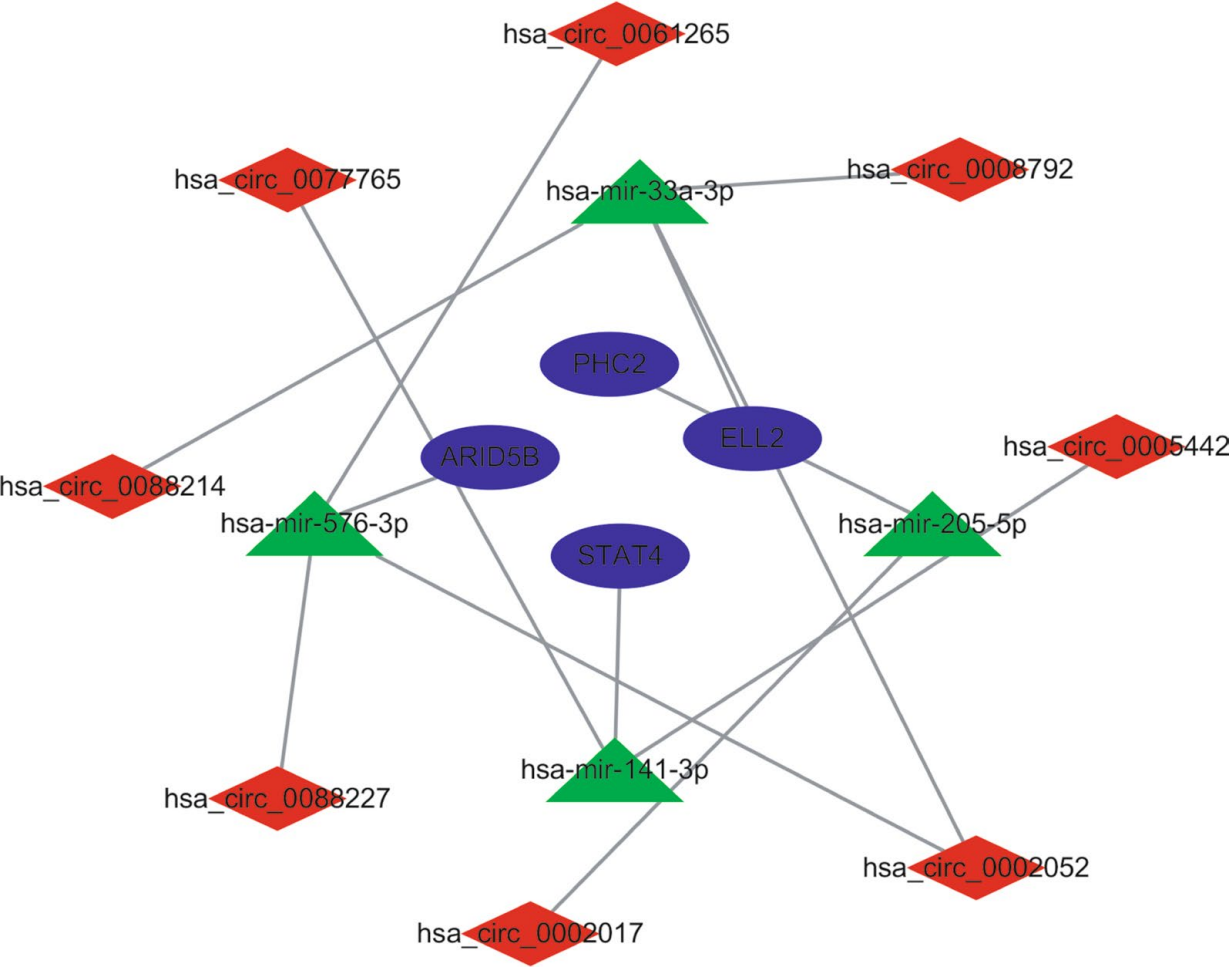
Construction of the circRNA-miRNA-mRNA subnetwork based on mRNAs with survival prognostic potential. Red indicates circRNAs, green indicates miRNAs, and blue indicates mRNAs

We downloaded OS mRNA expression profile data (101 samples) and clinical sample data from the TARGET database. We performed survival analysis for the 52 mRNAs in the constructed circRNA-miRNA-mRNA network. Four mRNAs (ARID5B, ELL2, PHC2, and STAT4) were significantly associated with survival prognosis in patients with OS (Fig. 7). We constructed a circRNA-miRNA-mRNA subnetwork with these four mRNAs (Fig. 8).

## Discussion

CircRNAs are a class of circular noncoding RNAs that have been observed in a variety of cancers [18–20], but their function is still unclear. Due to their special circular structure, circRNAs have high stability and great potential as new tumour markers [21]. Recent studies have found that circRNAs are involved in the development and progression of various cancers [22–24], such as gastric cancer, breast cancer, and colorectal cancer. Current evidence suggests that circRNAs can target miRNAs, often referred to as “miRNA sponges,” to reduce the levels of miRNAs and release their targeted inhibition of mRNAs, thereby regulating the expression of protein-coding genes [25–27]. These studies have found that circRNAs may play a key role in the development and progression of cancers.

OS is a common bone malignancy [1]. Current treatments for OS are mainly surgery and chemotherapy, but patient prognosis remains unsatisfactory [4]. Due to their stable structure, circRNAs may be a new strategy in the targeted therapy of OS [21]. A related study found that the circRNA LRP6 negatively regulates the levels of KLF2 and APC to promote the development of OS [28]. Dysregulated circRNA_100876 suppresses the proliferation of OS cancer cells by targeting microRNA-136 [29]. The upregulation of the circular RNA circ_0001721 predicts unfavourable prognosis in OS and facilitates cell cycle progression by sponging miR-569 and miR-599 [30]. Although these studies have found that circRNAs are dysregulated in OS and are involved in the development of cancer, the specific mechanism of circRNAs in OS remains unclear.

To investigate the role of circRNAs in OS, a circRNA-miRNA-mRNA regulatory network was constructed based on bioinformatics predictions and transcriptome data. GO enrichment analysis showed that genes in this network were mainly involved in the regulation of protein serine/threonine kinase activity, the transferase complex and ubiquitin protein ligase binding. Many studies have found that serine/threonine kinases play an important role in the occurrence and development of diseases. Insulin-like growth factor I (IGF-1) induces BNIP3 expression through a known AKT serine/threonine kinase 1 (AKT1)-mediated inhibitory phosphorylation of glycogen synthase kinase 3β (GSK-3β), which promotes cell growth and tumourigenesis [31]. Cooperative recruitment of Arl4A and Pak1 to the plasma membrane contributes to sustained Pak1 activation for cell migration [32]. Furthermore, the ubiquitination of proteins has been found to be involved in the cancer process. Suppression of the ubiquitin pathway by small molecule binding to ubiquitin enhances the doxorubicin sensitivity of cancer cells [33]. Epigenetic silencing of ubiquitin-specific protease 4 by snail1 contributes to macrophage-dependent inflammation and therapeutic resistance in lung cancer [34]. KEGG pathway analysis showed that the network was enriched in the PI3K-Akt signalling pathway, cell cycle and FoxO signalling, which have been confirmed by a large number of studies to participate in cancer progression [35–38]. These results suggest that the circRNAs in this network may play a key role in the occurrence and development of osteosarcoma. This result is worthy of further investigation.

To further study which circRNAs affect the prognosis of OS patients, we analysed the correlation between the expression levels of 52 mRNAs in this network and overall survival in OS patients based on TARGET data. Our study found that four mRNAs (ARID5B, ELL2, PHC2, and STAT4) may affect the prognosis of patients with OS. We constructed a circRNA-miRNA-mRNA subnetwork. There were 8 circRNAs (hsa_circ_0002052, hsa_circ_0088214, hsa_circ_0088227, hsa_circ_0008792, hsa_circ_0061265, hsa_circ_0002017, hsa_circ_00054442 and hsa_circ_0077765) and four miRNAs (miR-33a-3p, miR-205-3141, miR-576-3p) in the network. Studies have found that STAT4 is closely related to the prognosis of a variety of cancers, such as breast, liver, and stomach cancers [39–41]. By the targeting of ELL2 by microRNA-299 in glioblastoma multiforme, the upregulation of microRNA-299 is associated with a poor prognosis [42]. A previous study found that Phc2 controls the mobilization of bone marrow haematopoietic stem cells and progenitor cells by inhibiting Vcam1 expression [43]. ARID5B influences antimetabolite drug sensitivity and prognosis of acute lymphoblastic leukaemia [44]. Although we have identified four prognostic genes that have not been reported in OS, our predictions are consistent with studies in other cancers. Whether the dysregulation of these four genes will affect the prognosis of OS patients requires more in-depth research in the future.

At present, there are few reports on the mechanism of circRNAs in osteosarcoma. The novelty of this study is not only that the circRNA-miRNA-mRNA network was constructed through the GEO database but is also related to the prognosis of OS through the TARGET database. Previous studies identified OS survival prognostic genes based only on the GEO database, which contains few clinical samples of osteosarcoma. The TARGET database contains more osteosarcoma samples with clinical information, which can provide us with richer data for identifying genes related to survival prognosis. However, this study is also limited because it is mainly based on the analysis of sequencing data and may require further experimental exploration in the future.

## Conclusion

We identified differentially expressed circRNAs, miRNAs and mRNAs from publicly available microarray data to construct a circRNA-related ceRNA network. In addition, four mRNAs related to prognosis were identified based on the TARGET database. With these four mRNAs, we constructed a circRNA-miRNA-mRNA subnetwork that may be associated with prognosis in OS. The findings of this study may provide new insights into the pathogenesis of OS and suggest potential therapeutic targets that warrant further investigation.

## Supplementary information

**Additional file 1: Figure S1.** The expression levels of fifteen circRNAs in osteosarcoma.

**Additional file 2: Table S1.** Five hundred and three miRNAs predicted to target these 14 circRNAs.

**Additional file 3: Table S2.** A total of 136 miRNAs were upregulated in OS.

**Additional file 4: Table S3.** Twenty-four intersecting miRNAs.

**Additional file 5: Table S4.** A total of 324 potential target genes.

**Additional file 6: Table S5.** A total of 52 mRNAs were downregulated in OS in the mRNA expression profile data.

**Additional file 7: Table S6.** Fifty-two intersecting mRNAs.

## Data Availability

We declare that the data and materials in this study are provided free of charge to scientists for non-commercial purposes.
