# Supplementary figures and images for: Construction of a circRNA-miRNA-mRNA network based on competitive endogenous RNA reveals the function of circRNAs in osteosarcoma
Source: Cancer Cell Int. 2020 Feb 10;20:48. doi: 10.1186/s12935-020-1134-1 (PMC7011443; doi:10.1186/s12935-020-1134-1)

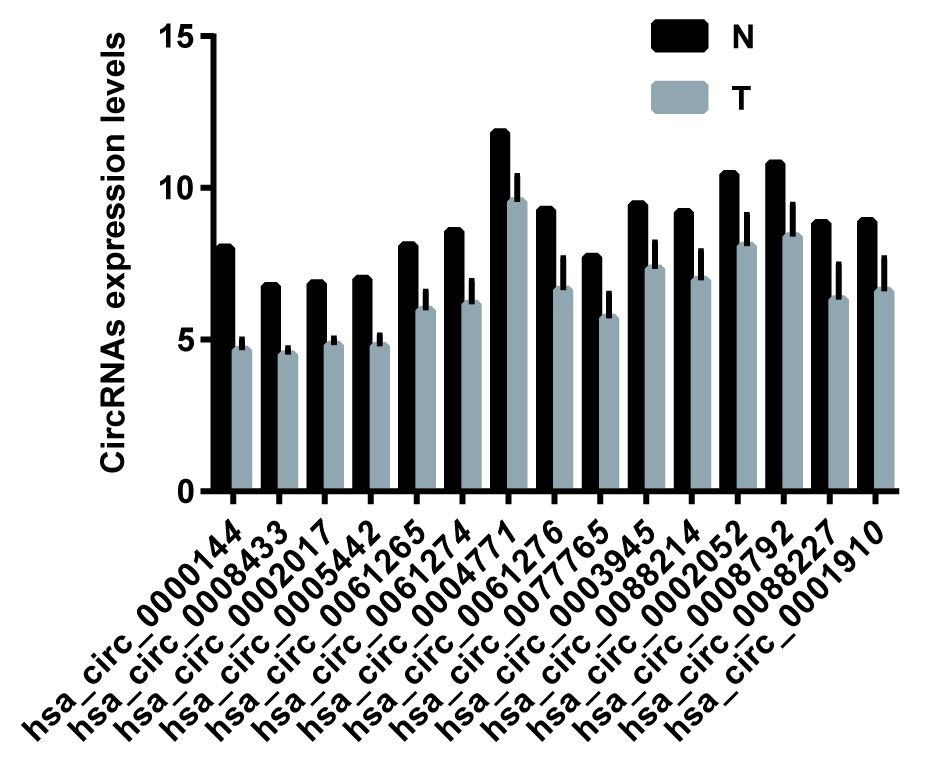

Supplement: Supplementary file 1 — Additional file 1: Figure S1. The expression levels of fifteen circRNAs in osteosarcoma. [file 12935_2020_1134_MOESM1_ESM.tif]
